# Novel Profiles of Family Media Use: Latent Profile Analysis

**DOI:** 10.2196/59215

**Published:** 2025-03-06

**Authors:** Nicole Hamp, Jenny Radesky, Heidi M Weeks, Alison L Miller, Niko Kaciroti

**Affiliations:** 1 Division of Developmental Behavioral Pediatrics, University of Michigan Medical School, D2204 CS Mott Children’s Hospital, 1500 E. Medical Ctr Dr, Ann Arbor, MI, 48109-5718, United States, 1 2485615770; 2 University of Michigan School of Public Health, Ann Arbor, MI, United States

**Keywords:** preschool, child, digital media, mobile media, media use, latent profile analysis, computer use, LPA, technology use, survey, questionnaire, pediatrics

## Abstract

**Background:**

Over the past 3 decades, digital and screen media have evolved from broadcast, stationary platforms to a complex environment of interactive, omnipresent, mobile media. Thus, clinical guidance centered around unidimensional concepts such as “screen time” must be modernized to help families navigate the intricate digital ecosystems of readily available entertainment and information.

**Objective:**

This study aimed to identify and examine distinct latent profiles of media use in families with young children. We hypothesized that latent profile analysis (LPA) would identify different media use profiles characterized by more heavy, reactive, individual, and permissive media use and more intentional, regulated, or shared uses of media.

**Methods:**

We analyzed data from 398 preschool-aged children. English-speaking parents were recruited through community settings. Participants completed surveys regarding several aspects of family media use, such as child device use or activities, parent concerns and attitudes, limit setting and mediation, parent media use, and technology interference, examined in an LPA. The number of latent media profiles was determined using Bayesian Information Criteria. Parents also completed validated scales of parenting stress, depression symptoms, parenting style, child behavior, child sleep, and household disorganization. Multivariable logistic regression was used to examine parent, child, and household predictors of group membership.

**Results:**

The LPA yielded 2 distinct groups that differed in the duration of media used by parents and children, to calm children or help them fall asleep. Statistically significant differences between groups included: families in group 1 (n=236, which we termed social-emotional drivers) had parents who preferred interactions via text or email to in-person (*P*=.01) and were more likely to use media to calm their children (*P*=.03); in contrast, families in group 2 (n=162, intentional media) used more task-oriented media, like audio and nongame apps (*P*=.01), had more concerns about effects of media on child language development (*P*=.04), and used more media restrictions (*P*=.01). In regression models, female sex of the parent respondent, greater number of siblings, and later child sleep midpoint independently predicted group 1 membership.

**Conclusions:**

Findings suggest divergent family media use patterns that can be categorized into 2 main media user groups: those using media to buffer social situations or regulate emotions and those planning mobile device use around functional purposes and concerns around media exposure. Profiles were associated with household size and child sleep. More research is needed to examine the impact of social and emotional uses of media on child outcomes.

## Introduction

### Background

The landscape of digital technology use has changed dramatically over the past few decades. Digital and screen media have evolved from broadcast, stationary platforms, where screens stay put, plugged into the wall, and messages are transmitted broadly in a one-to-many model, to a world of interactive, mobile media, where screens can follow users wherever they go and interact in a bidirectional manner. For this reason, researchers have questioned whether clinical guidance centered around unidimensional concepts such as “screen time” are helpful to parents trying to navigate digital ecosystems of readily available entertainment and information [1], particularly considering families’ increased technologic dependence during and following the COVID-19 pandemic.

Distinctions between traditional (eg, television [TV]) and mobile, interactive media are important for several reasons. First, the portability and easy accessibility of mobile media inherently allows for more spontaneous and reactive use patterns in which the technology becomes increasingly integrated into daily routine and activities [2,3]. Second, small, handheld screens are more difficult for parents to monitor [4]. And third, mobile media use has rapidly become exceedingly common, even in infants and toddlers. As of 2017, 98% of homes of children 0‐8 years old had a mobile device, and one third of all screen time in that same age group, who use on average almost 2.5 hours of screen media per day, was mobile [5].

### Previous Work

In light of modern technologic advances, new ways of studying, conceptualizing, and framing media use guidance have been proposed. Young children’s media use has been conceptualized as the “3 Cs”, that is, content, context, and the individual child given the important role each of these factors plays in shaping child responses to media [6]. However, pervasive use of mobile media by families with young children requires new concepts such as use of devices for on-demand calming and keeping children occupied during daily activities.

To capture holistic patterns of family media use, it is also important to consider parents’ mobile device use, which interrupts parent-child interaction [7] and is associated with less responsiveness [8], but is an important part of parent social connection, work-life, and day-to-day functioning [9]. Parents’ mediation behaviors (practices such as coviewing, teaching children about media content, or setting limits) also shape children’s responses to media [10]. Finally, child and parent media use are highly correlated [11], yet are usually studied in isolation. One previous attempt to describe family-level media behaviors [12] primarily focused on viewing duration and type of media use, rather than the several contextual variables or social-emotional drivers of media use in the current digital environment.

### Goal of This Study

The current study aims to identify patterns that include the aforementioned concepts, examined through latent profile analysis, to try to identify patterns and concepts that might generate insights for clinical guidance and future research. Specifically, we sought to identify novel patterns of family media use that consider child duration and frequency of media activities; child use to keep occupied, regulate behavior, or fall asleep; parent attitudes about child use; limit setting and mediation; parent media use; and “technoference” (ie, technology interference in parent-child activities). We hypothesized that latent profile analysis (LPA) would identify different media use profiles characterized by more reactive, heavy, individual, and permissive media use; and more intentional, regulated, or shared uses of media. We examined these patterns and their associations with parent, child, and household characteristics within a large cohort of preschool-aged children, as early childhood is an important time of establishing media use habits [13].

## Methods

### Overall Study Design

We analyzed data from the Preschooler Tablet Study, a longitudinal cohort study (NICHD R21HD094051) examining associations between early childhood digital media use and social-emotional development. The present analysis used REDCap (Research Electronic Data Capture) [14] and Qualtrics survey data from the baseline data collection wave (August 2018-May 2019).

### Ethical Considerations

The study was approved by the University of Michigan Institutional Review Board (HUM00131980). Parents provided electronic informed consent for themselves and on behalf of their young children. Participants were informed that they could opt out of the study at any time. Data downloaded from REDCap and Qualtrics were stored on secure password-protected servers at the University of Michigan. Data was not de-identified prior to analysis; all participants were assigned a study ID number that only linked to identifying information on REDCap, a HIPAA-secure database to which only approved study personnel had access. Participants received $40 for completing data collection procedures.

### Participants

Parents of young children were recruited through flyers posted in community centers, preschools, childcare centers, and pediatric clinics in southeast Michigan, as well as our university’s online participant registry and social media advertisements. Interested parents who contacted the study team were emailed a link to an eligibility questionnaire. Eligibility criteria is shown in Textbox 1. To improve generalizability, participating children did not need to regularly use mobile devices to be included in the study.

Textbox 1. **Study inclusion and exclusion criteria.**

**Inclusion criteria:**

- Parent was legal guardian of a 3‐ to 4.99-year-old child.
- Parent lived with the child at least 5 days per week.
- Parent understood English sufficiently enough to complete questionnaires and provide consent.
- The family owned at least 1 Android or iOS tablet or smartphone.

**Exclusion criteria:**

- Child developmental delays.
- Use of psychotropic medication.

### Survey Measures: Child, Parent, and Household Characteristics

After providing electronic informed consent, respondent parents completed web-based surveys with a variety of questionnaires to assess characteristics of the child, parent, and household, as well as family media use practices. Demographic characteristics were collected for children’s age, sex, race, ethnicity (investigator-defined categories shown in Table 1), daycare or preschool enrollment, average sleep pattern (sleep onset and wake time, from which duration and midpoint were calculated, as well as sleep latency and overnight awakenings), prematurity, and whether they were an only child; parent age, gender, educational attainment, marital status, and employment status. We also used validated questionnaires to assess parent depression symptoms (Centers for Epidemiologic Studies-Depression Scale) [15], parenting stress (Parenting Stress Index-Short Form) [16], and parenting styles (laxness and harshness subscales of The Parenting Scale) [17]; as well as household income, size, composition, and disorganization (Chaos, Hubbub, and Order Scale) [18]. Child self-regulation abilities were assessed with the Emotional Reactivity subscale of the Child Behavior Checklist-Preschool [19], the Surgency subscale of the Rothbart Child Behavior Questionnaire-Very Short Form [20], and the Behavior Rating Inventory of Executive Function-Preschool [21].

**Table 1.** Participant sociodemographic characteristics.

Characteristics			Values
Parent			
	Age, mean (SD)		34 (4.7)
	Sex, n (%)		
		Male	25 (6.3%)
		Female	373 (93.7%)
	Education, n (%)		
		≤High school or GED^a^	25 (6.3%)
		Some college or a 2-year degree	126 (31.7%)
		4-year college degree	100 (25.1%)
		Advanced degree	147 (36.9%)
	Marital status, n (%)		
		Married or has a partner	360 (90.9%)
		Single, separated, or divorced	36 (9.1%)
	Employment, n (%)		
		Unemployed	110 (27.6%)
		Part-time	76 (19.1%)
		Full-time	185 (46.5%)
		Multiple jobs	27 (6.8%)
	Scales, mean (SD)		
		Depression symptoms (CES-D^b^ score)	9.32 (8.87)
		Parenting Stress Index percentile	44.6 (32.9)
		Parenting Scale – Laxness Subscale	2.61 (0.76)
		Parenting Scale – Overreactivity Subscale	2.56 (0.74)
Child			
	Age, mean (SD)		3.85 (0.54)
	Sex, n (%)		
		Female	186 (46.7%)
		Male	212 (53.3%)
	Race/ethnicity, n (%)		
		Asian or Pacific Islander	11 (2.8%)
		Black or African American, non-Hispanic	20 (5.1%)
		Hispanic, any race	26 (6.6%)
		Multiple races, non-Hispanic	32 (8.1%)
		Native American or Alaska Native	5 (1.3%)
		White, non-Hispanic	302 (76.3%)
	Only child, n (%)		
		Yes	69 (17.3%)
		No	329 (82.7%)
	Child gestational age, n (%)		
		<37 weeks (premature)	32 (8%)
		37 weeks or later	366 (92%)
	Child preschool or child care, n (%)		
		Center-based child care	250 (65.8%)
		Home-based child care	30 (7.9%)
		Stays home with parent or caregiver	100 (26.3%)
	Sleep, mean (SD)		
		Sleep duration	10.8 (0.8)
		Sleep midpoint (number of hours after 12 AM)	1.87 (0.82)
		Sleep latency >30 min	106 (26.6)
		Overnight awakenings	226 (60.4)
	Scales, mean (SD)		
		CBQ-VSF^c^ Surgency Subscale	4.40 (0.86)
		BRIEF-P^d^ General Executive Composite	49.2 (12)
		CBCL-P^e^ – Emotional Reactivity Subscale	3.69 (2.82)
	Household, mean (SD)		
		Income-to-needs ratio	2.95 (1.71)
		CHAOS^f^ score	3.29 (2.93)

a GED: General Educational Development.

b CES-D: Centers for Epidemiologic Studies-Depression

c CBQ-VSF: Child Behavior Questionnaire Very Short Form

d BRIEF-P: Behavior Rating Inventory of Executive Function-Preschool

e CBCL-P: Child Behavior Checklist–Preschool

f CHAOS: Chaos, Hubbub, and Organizational Scale

### Survey Measures: Media Use

Parents also completed a 75-item questionnaire about family media use derived from the CAFE (Comprehensive Assessment of Family Exposure) Consortium Qualtrics Survey, which has been described elsewhere [22]. This survey asks about technology and device ownership, content and context of media use, parent media use, and mediation practices (refer to Textbox 2 for constructs assessed). Questions on the survey addressed types of devices in the home and locations of those devices, parent attitudes toward media and concerns regarding child use of media, duration of use on weekdays versus weekends, time of use and environmental context of use (for example while falling asleep or while in transit), usual content (for example streaming video versus playing games), family interactions around media, and media-use functions.

Textbox 2. **Media-related constructs assessed through the CAFE (Comprehensive Assessment of Family Exposure) questionnaire.**

**A. Child ownership and frequency of activities**

A1. Child ownership of mobile media device

A2. Child keeps device in bedroom

Frequency of mobile device use for specific activities:

(A3. Watch TV; A4. Watch movies; A5. Play games; A6. Use apps that are not games; A7. Read electronic books; A8. Listen to music or audiobooks; A9. Take photos; A10. View photos/videos)

**B. Child instrumental or regulatory uses of media**

B1. Use of media during travel in car or public transit

B2. Use of TV to calm when upset

B3. Use of mobile devices to calm when upset

Use of all types of screen media by parent for specific purposes related to child:

(B4. To educate child; B5. Calm child down; B6. Keep child busy; B7. Communicate with family and friends; B8. Because child enjoys it)

B9. Use of devices at bedtime

B10. Use of devices while falling asleep

**C. Parent media knowledge and attitudes**

Parent concerns that child will:

(C1. Be exposed to inappropriate content; C2. Become inattentive as a result of using screen media; C3. Become addicted to screen media; C4. Miss out on other important opportunities that are more valuable than screen media; C5. Be exposed to harmful electromagnetic waves; C6. Have poorer language development).

**D. Mediation strategies**

Presence of media content limits:

(D1. Parents blocks specific media content on TV/devices; D2. Parent uses web blockers/controls; D3. Parent only allows child to watch “child-friendly” content; D4. Parent uses ratings to decide what child will watch; D5. Child media use only allowed if parent is in the room).

D6. Media time limits are consistently enforced

D7. Media content limits are consistently enforced

D8 – D22: Valkenburg Mediation Scale (Social Coviewing, Instructive Mediation, and Restrictive Mediation)

**E. Parent media use**

Outside of work hours, parent feels:

(E1. The need to stay connected to work almost constantly; E2. The need to stay connected to friends and social media almost constantly; E3. It is easy to multitask between children and using a phone or mobile device; E4. Sometimes overwhelmed by how much they have to do on their phone or mobile device; E5. That they prefer to interact with others via texting, email, or social media, rather than in person; E6. Using their phone or mobile device allows them to “escape” a little bit while they’re with their children; E7. Sometimes “addicted” to mobile media like smartphones or tablet devices).

Frequency of specific activities during a typical weekday (Monday-Friday):

(E8. Watch TV; E9. Use the computer; E10. Read traditional books; E11. Read electronic books; E12. Play videogames on console game player; E13. Use an iPad, iTouch, or similar device (not including a smartphone); E14. Use a smartphone for things like texting, playing games, watching videos, checking email, or surfing the internet).

Frequency of specific activities during a typical weekend day (Saturday-Sunday):

(E15. Watch TV; E16. Use the computer; E17. Read traditional books; E18. Read electronic books; E19. Play videogames on console game player; E20. Use an iPad, iTouch, or similar device (not including a smartphone); E21. Use a smartphone for things like texting, playing games, watching videos, checking email, or surfing the internet).

**F. Technoference**

Frequency of parent phone use during specific activities:

(F1. During meals; F2. While getting child(ren) ready for school; F3. During playtime; F4. During bedtime routine; F5. While driving child(ren) to or from activities or when riding on public transportation; F6. At the playground).

### Data Analysis

Of the 423 participants who provided consent and completed surveys, we excluded participants who did not complete (n=19) or had substantial missing data (n=6) on the media use questionnaire. This left 398 participants in this study available for the LPA. All media variables were included in LPA, a person-centered statistical method to identify distinct groups of participants with similar median profiles within each group. Using Bayesian Information Criteria (BIC), the LPA with the lowest BIC value yielded 2 distinct groups.

Wilcoxon Mann-Whitney tests were used to compare media use questionnaire items between the groups identified by the LPA. Then separate multivariable logistic regression models were built to estimate the odds of being in group 1 versus group 2 for each set of parent (Model I), child (Model II), and household (Model III) predictors. As our approach was exploratory, we started with including all parent, child, or household characteristics in each respective model and conducted backward elimination, resulting in the most parsimonious model that retained only variables showing significant associations at a *P* value of <.05. For all characteristics significantly associated with group membership in Models I, II, or III, we built a combined Model IV to test which characteristics were independently associated with group membership.

## Results

### Participant Demographics

Parents were 93.7% female (373/398), 34 (SD 4.7) years old, and 62% (247/398) had a 4-year college degree or more; children were 3.8 (SD 0.54) years old, 76.3% (302/398) were White and non-Hispanic, and 82.7% (329/398) had siblings in the household (Table 1).

### Evaluation Outcomes

**Figure 1.**
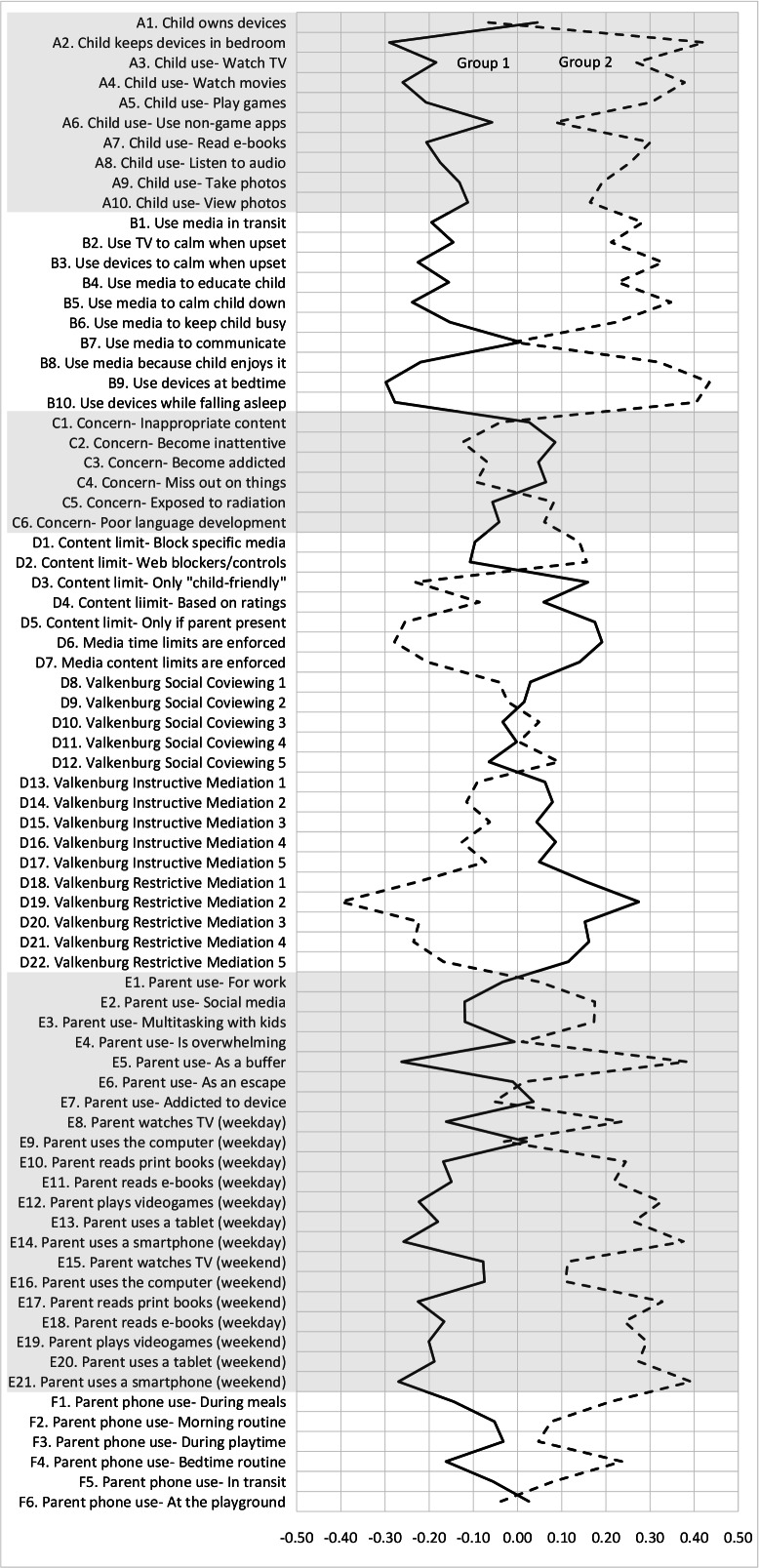
Latent Profile Analysis: media use profiles. Standardized means by variable for group 1 versus group 2. Lettering describes variable type: A. child ownership and frequency of activities; B. child instrumental or regulatory uses of media; C. parent media knowledge and attitudes; D. mediation strategies; E. parent media use; F. technoference.

Latent profile analysis yielded 2 distinct groups of media users (Figure 1). Families in group 1 (n=236) were more likely to prefer interactions through text, email, or social media rather than those in person (*P*=.01) and more likely to use TV shows or DVDs to calm their children (*P*=.03). Parents in group 1 used their mobile device more frequently during the week to read electronic books (*P*=.04). In contrast, group 2 (n=162) used more task-oriented media, including more audio and nongame apps (*P*=.01), had more concerns about effects of media on language development (*P*=.04), and used more media restrictions (*P*=.01).

As shown in Figure 1, several additional variables approached significance (*P*<.20) that warrant mention. Parents in group 1 were more likely to feel overwhelmed by how much they have to do on their phone or mobile device (*P*=.12) and reported that using the phone or mobile device allowed them to “escape” a little bit while with their children (*P*=.14). They were more likely to watch TV or DVDs (*P*=.15) or use the computer (*P*=.10) over the weekend than were families in group 2. Group 1 families also reported using more content restrictions for what their children see in the media with internet filters, parental controls, or apps to block certain websites (*P*=.11) , as well as use of parental media websites (eg, common sense media) to decide what types of programs are appropriate for their child. Finally, group 1 families were more likely to use their mobile device to take photos (*P*=.11).

Group 2 families preferred using their mobile devices to view photos or home videos (*P*=.13) in addition to the other task-oriented media described above. They also noted concerns that children will become inattentive as a result of using screen media (*P*=.11) and more frequently restrict the amount of child viewing (*P*=.06).

In logistic regression models (Table 2), the only parent characteristic that was significantly associated with group 1 membership (vs group 2) in Model I was female parent sex. In Model II, children with longer duration of sleep had lower odds of group 1 membership, while those with later sleep midpoint and prematurity showed increased odds of group 1 membership. Households with more siblings had a borderline increased odds of group 1 membership in Model III. With all characteristics considered in the same model (IV), independent associations remained for female parent sex, greater number of siblings, and later child sleep midpoint.

**Table 2.** Multivariable logistic regression models predicting group assignment.

Model and variable		Group 1 (social-emotional drivers) versus group 2 (intentional media), aOR^a^ (95% CI)
Model I: parent characteristics		
	Parent sex (male vs female)	0.36 (0.16-0.84)
Model II: child characteristics		
	Sleep duration (per 1 hour)	0.72 (0.55-0.95)
	Sleep midpoint (per 1 hour)	1.5 (1.13-1.98)
	Prematurity (no vs yes)	2.26 (1.06-4.8)
Model III: household characteristics		
	Number of siblings (per sibling)	1.23 (0.998-1.5)
Model IV: all characteristics		
	Parent sex (male vs female)	0.3 (0.12-0.76)
	Number of siblings (per sibling)	1.27 (1.02-1.57)
	Sleep midpoint (per 1 hour)	1.51 (1.1-2.07)

a aOR: adjusted odds ratio

## Discussion

### Principal Findings

This study used a wide range of questions about child, parent, and household context of media use to identify coherent patterns of media use that are relevant to pediatric research or clinical intervention. Latent profile analysis results suggest that people may be predisposed to different media-use patterns based on individual motivations. Group 1 preferred text and email interactions to those in-person and used media to calm their children. These behaviors may be interpreted as use of media based on social-emotional drivers. In contrast, group 2 used media for more functional purposes. This group preferred more nongame and audio applications. They seemed warier of media, placed more restrictions around child media use, and had more concerns about the effect of media on child development. Though these findings in some way confirmed our initial hypothesis, that some types of media users are predisposed to more reactive-use patterns (group 1), while others are more predisposed to intentional and regulated uses of media (group 2), the tendency to use media as a sort of social-emotional buffer was not a factor we considered in our initial hypothesis.

When examining the overall patterns of media use between groups, a few theoretically coherent concepts arise. In group 1, described as using media based on social-emotional drivers, there appeared to be more parent use of media as an “escape” from childrearing demands, such as more parental media during the weekends, which is typically time families are together during the day. In previous qualitative work, parents have described using mobile devices and social media as a “virtual escape” when their child stresses them out [9], when they want to avoid parenting tasks [23], or when intentionally not wanting to engage with difficult child behavior [24]. Furthermore, compared with parents in group 2 who were more likely to view, but not take, photos or home videos on a mobile device, group 1 families took photos on their device more frequently, an action that by definition interrupts a social moment and introduces a physical barrier between the individual taking the photograph and the subjects.

In group 2, parent media use appeared more goal oriented, and more limits and restrictions were placed on child media use, which may be related to greater concerns about media’s effects on child wellbeing. This pattern of device usage has been described as “instrumental” (ie, goal directed and purposeful) rather than “ritualistic” in previous work [25], and is hypothesized to be related to the individual motivations for engaging with technology. In this study, we describe this pattern of device usage as intentional, similarly noting that this type of media use is meant to fulfill a purpose rather than for pleasure or distraction. Though we did not observe increased odds of group 2 membership based on measures of parental mental health or child behavior, a recent study using latent class analysis found stronger well-being indicators for “family-engaged adolescents” who live in families with family-owned devices, positive parent relationships, and lower parental social media use [26]. Higher wellbeing also occurred in teens who placed lower importance on technology and were expected to follow household technology rules. Future research may therefore examine the relationship between these multiple classes of media users in a longitudinal manner to determine if “intentional” media-use families who set early boundaries around child media use are more likely to have “family-engaged adolescents” with better social-emotional outcomes.

It is surprising that socioeconomic status, parenting stress, household disorganization, and child behavioral difficulties were not associated with membership in group 1. In previous research, longer screen time duration and higher parent technology interference have been linked with higher parenting stress [27-29]. Recent work has also suggested that children’s screen time is a marker of family distress due to multiple psychosocial factors [30]. However, these studies only examined the variable of screen time, while our approach identified larger family media use patterns that appear independent of socioeconomic factors in this cohort.

We did find that mothers are more likely to use media as a social-emotional buffer and that this type of media use is more common in larger families. It is possible that mothers or parents of larger families may experience higher caregiver burden and, as a result, are using media for more self-regulatory purposes and to calm or manage child behavior more frequently. Indeed, use of digital technology as a “babysitter,” to provide caregiver respite or allow parents time to tend to other tasks, is a concept that is well-described in research literature and mainstream news, albeit with some differences in acceptance across cultures [31-35]. Evidence suggests that use of media to occupy children may be especially relevant in homes where children require more attention or behavioral management due to temperament differences [36], or where there is limited support for the primary caregiver. One study found that parents who lack support from a partner or who are uncertain about their parenting skills were more likely to use media as a distractor and concluded that “media are thus especially used as a distractor in the family when parents feel that it is difficult to keep the household going by themselves” [34].

Another correlate of group membership was later sleep midpoint (ie, the calculated midpoint between reported average sleep onset and wake time), with group 1 having later sleep midpoints than group 2. This may be explained by the fact that group 2, despite any significant difference in overall parenting style, seemed more prone to limit setting. What is perhaps most surprising about our study findings are the variables that did not predict group membership including parent education, marital status, employment, and child behavior variables such as emotional reactivity and surgency. Although human-computer interaction research has identified individual predictors of smartphone usage habits such as personality [37], attachment style [38], and executive functioning [39], we found no associations of parenting style (such as laxness vs harshness), parenting stress, or depression symptoms with group membership.

### Limitations

Our study was limited in generalizability due to our study population which included mostly White, non-Hispanic, higher-educated, and female-parent responders. While our cohort reflected the racial and ethnic diversity of our local area, results may not be generalizable to other populations. In addition, the data we analyzed on media use was all from self-report questions, which can lead to single-reporter bias. We also reported on several associations that did not achieve significance, but were near significant, that we included in our results as we felt the data helped to demonstrate an overall trend. Greater insight into the reasons for media use may have been gleaned from a mixed methods approach, where follow up semistructured interviews could have explored themes related to media as a social-emotional buffer versus to fulfill a desired goal.

### Conclusions

Results of our study suggest that people likely do have different motivations behind their use of digital media that may be reflected in their usage patterns. The significance of these different media usage patterns for the long-term outcomes of children and families is yet to be determined. It is possible, and in fact likely, that each pattern of media use may be considered adaptive in certain situations and maladaptive in others. By having a better understanding of why and how different families use media in their daily lives, pediatric care providers can provide more individualized anticipatory guidance regarding technology use by the whole family, including limit setting, use of media for calming, and how devices impact family dynamics. For example, by understanding that a parent is more prone to using mobile media to calm their child, a pediatrician might suggest that such a parent reflect on the frequency with which they use such calming techniques to ensure that they are also providing their child opportunities to practice frustration tolerance using techniques that go beyond distraction with media.

The research implications of our study may allow us to classify the media use patterns of families to better examine the long-term effects of media use on child health and development. Follow up studies could examine trajectories of profiles over childhood to determine their stability and how they relate to child outcomes over time. Future research directions should also include nationally representative populations, objective device use data, or reports from multiple household members (eg, parents and children).

## Abbreviations:

BIC: Bayesian Informed Criteria
CAFE: Comprehensive Assessment of Family Exposure
DVD: digital versatile disc
LPA: latent profile analysis
REDCap: Research Electronic Data Capture
TV: television
